# Evaluation of exosomal non-coding RNAs in cancer using high-throughput sequencing

**DOI:** 10.1186/s12967-022-03231-y

**Published:** 2022-01-15

**Authors:** Kamran Hosseini, Maryam Ranjbar, Abbas Pirpour Tazehkand, Parina Asgharian, Soheila Montazersaheb, Vahideh Tarhriz, Tohid Ghasemnejad

**Affiliations:** 1 Department of Molecular Medicine, Faculty of Advanced Medical Sciences and Technologies, Shiraz University of Medical Sciences, Shiraz, Iran; 2 Student Research Committee, Shiraz University of Medical Sciences, Shiraz, Iran; 3 Present Address: Department of Medical Genetics, Faculty of Medicine, Tabriz University of Medical Sciences, Tabriz, Iran; 4 Department of Biochemistry and Clinical Laboratories, Faculty of Medicine, Tabriz University of Medical Sciences, Tabriz, Iran; 5 Department of Pharmacognosy, Faculty of Pharmacy, Tabriz University of Medical Sciences, Tabriz, Iran; 6 Drug Applied Research Center, Tabriz University of Medical Sciences, Tabriz, Iran; 7 Molecular Medicine Research Center, Biomedicine Institute, Tabriz University of Medical Sciences, Tabriz, Iran

**Keywords:** Exosome, Nucleic acids, Biomarkers, NGS, Cancer, ncRNAs

## Abstract

Clinical oncologists need more reliable and non-invasive diagnostic and prognostic biomarkers to follow-up cancer patients. However, the existing biomarkers are often invasive and costly, emphasizing the need for the development of biomarkers to provide convenient and precise detection. Extracellular vesicles especially exosomes have recently been the focus of translational research to develop non-invasive and reliable biomarkers for several diseases such as cancers, suggesting as a valuable source of tumor markers. Exosomes are nano-sized extracellular vesicles secreted by various living cells that can be found in all body fluids including serum, urine, saliva, cerebrospinal fluid, and ascites. Different molecular and genetic contents of their origin such as nucleic acids, proteins, lipids, and glycans in a stable form make exosomes a promising approach for various cancers’ diagnoses, prediction, and follow-up in a minimally invasive manner. Since exosomes are used by cancer cells for intercellular communication, they play a critical role in the disease process, highlighting the importance of their use as clinically relevant biomarkers. However, regardless of the advantages that exosome-based diagnostics have, they suffer from problems regarding their isolation, detection, and characterization of their contents. This study reviews the history and biogenesis of exosomes and discusses non-coding RNAs (ncRNAs) and their potential as tumor markers in different types of cancer, with a focus on next generation sequencing (NGS) as a detection method. Moreover, the advantages and challenges associated with exosome-based diagnostics are also presented.

## Background

The gold standard for solid tumors' diagnosis is surgical tissue biopsies usually done after a series of imaging scans [1–3]. The time, cost, repeatability, accessibility, and invasiveness of tissue biopsy-based diagnostics make them less desirable, leading to a late-stage diagnosis [4–7]. For cancer patients to achieve the best possible treatment outcome, it is imperative to develop non-invasive and cost-efficient diagnostic methods that have the potential for early tumor detection and monitoring while the patient is undergoing treatment [3]. Liquid biopsy and molecular profiling of biofluids have gained increasing attention in recent decades due to their less invasive approaches, real-time tumor status insights, low cost, and ability to address tumor heterogeneity [8, 9]. The presence of biological materials in biofluids enables researchers to detect tumors occurring in distant tissues that are not symptomatic enough to be detected by conventional imaging techniques [10]. In fact, cancer care has been revolutionized through liquid biopsy, which has enabled early detection, improved diagnosis, predicted and determined therapeutic outcomes, and directed precision medicine [9]. Based on the source of tumor-derived constituents in biofluids, liquid biopsies for cancer are categorized into: (i) extracellular vehicles (EVs) and tumor-derived exosomes, (ii) circulating tumor cells (CTCs), and (iii) circulating tumor DNA (ctDNA) [8, 9]. Exosomes with sizes between 30 and 150 nm are small extracellular vesicles comprised of a lipid bilayer membrane and are responsible for intercellular communication. Stemming from the endosomal pathway within their parental cells, exosomes enclose a wide range of molecules including proteins, lipids, deoxyribonucleic acid (DNA), and different kinds of ribonucleic acids (RNAs) [10]. These contents are important for intercellular communication, both in normal physiological processes as well as in pathological conditions such as immune responses, pregnancy disorders, and cancer [11]. The presence and the stability of exosomes in most body fluids and their contents' similarity to parental cells make them promising candidates for developing new approaches for cancer diagnosis [12, 13]. Using high-throughput sequencing technologies, researchers have found that exosomes comprise different RNA populations including messenger RNAs (mRNAs), transferred RNAs (tRNAs), ribosomal RNAs (rRNAs), microRNAs (miRNAs), small nuclear RNAs (snRNAs), circular RNAs (circRNAs), long non-coding RNAs (lncRNAs), and P-element-induced wimpy testis (PIWI)-interacting RNAs (piRNAs) [14–16]. The current review discusses the history and biogenesis of exosomes, NGS technology, and the potential ncRNAs biomarkers in different types of cancer detected by NGS. Furthermore, a brief overview of the advantages and challenges of exosome-based diagnostic techniques is provided.

## The discovery of exosomes

About four decades ago, Pan, Stahl, and Johnstone discovered EVs while studying the loss of transferrin during reticulocyte maturation in blood. They believed that these vesicles are sprouted from different parts of the plasma membrane of cultured cells [17–20]. Another group of researchers found that EVs result from germination into the intracellular endosome, which eventually forms multivesicular bodies (MVBs). MVBs produce intraluminal vesicles (ILVs) called exosomes. After a while, it turned out that exosomes are involved in intercellular communication [21, 22].

## Biogenesis, secretion, and content of exosomes

**Fig. 1:**
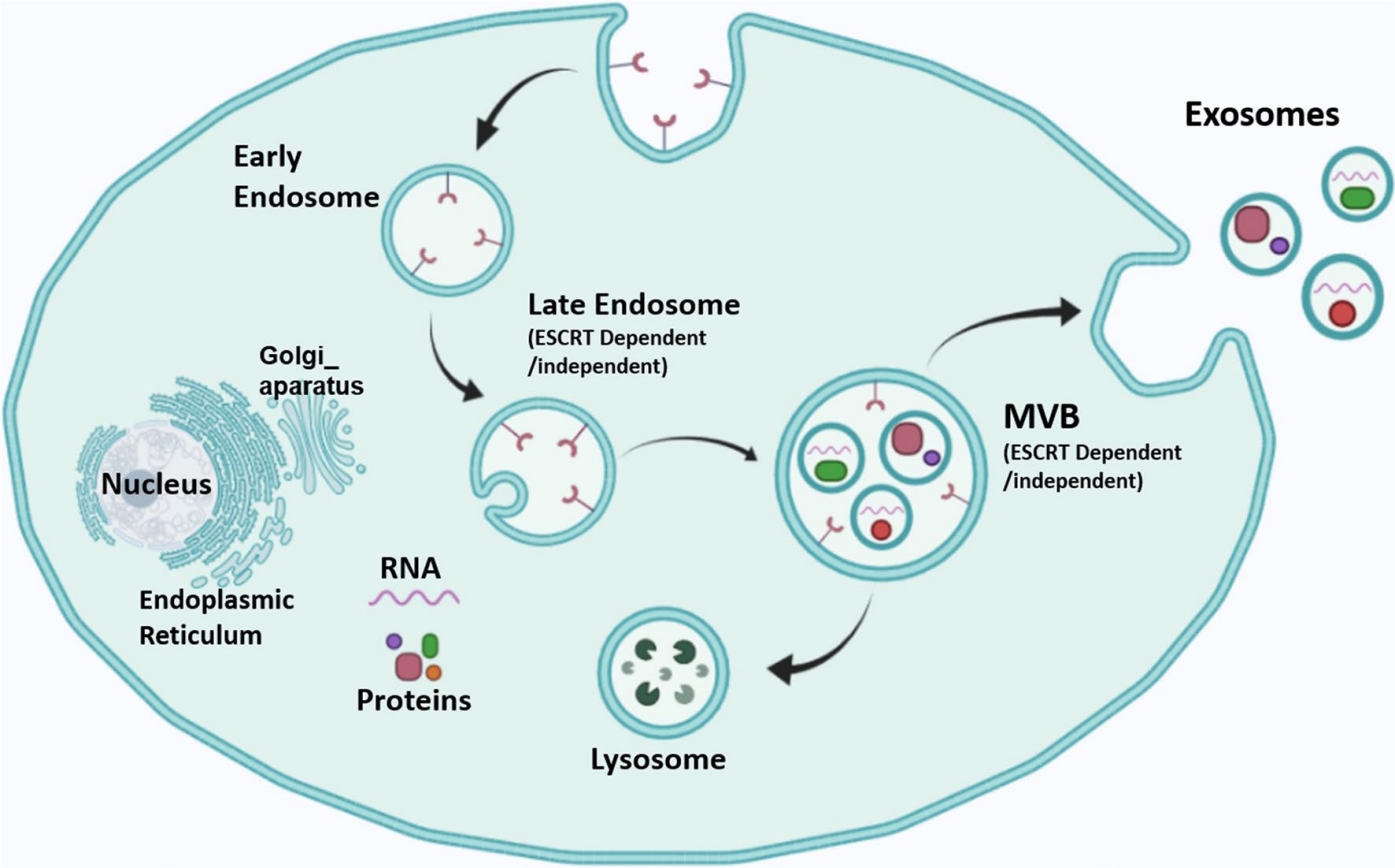
Biogenesis mechanism of exosomes, which starts with the development of the endosome and then forms multivesicular bodies (MVBs). MVBs can both integrate with lysosomes for degradation or fuse with the cellular membrane to release exosomes. These processes can happen by both ESCRT-dependent and ESCRT-independent pathways

Exosomes with endocytic origin are morphologically very small vesicles whose structure is rich in macromolecules such as proteins and nucleic acids [10, 23]. Their biogenesis involves several stages of a complex biological process that have not yet been fully and accurately determined. The first processing stage begins with the lipid raft domains of the plasma membrane. The primary intracellular endosomes are obtained by budding into the membrane and then turning into the final endosomes via the Golgi apparatus. During this process, intraluminal vesicles are formed through the ESCRT-dependent mechanism (endosomal sorting complexes required for transmission) or non-ESCRT-dependent mechanisms within the final endosomes (Fig. 1). During the budding process of the endosomal membrane, biomolecules are accumulated inside them and form MVBs [24, 25]. As mentioned, ESCRT mediates the process of biogenesis [17] and contains a total of 20 proteins that are generally divided into four categories of ESCRT-0,-I,-II,-III [26, 27]. ESCRT-0 binds to ubiquitinated proteins in the endosomal membrane [28]. ESCRT-I and -II are responsible for membrane deformation in the form of buds with consecutive cargo [27], and ESCRT-III is responsible for the vesicular incision [27, 29]. Under physiological and pathological conditions, exosomes can be secreted from a variety of cancer cells [30, 31], platelets [32], mast cells [30], dendritic cells [33], astrocytes [34], B and T cells [35], etc. Numerous studies have shown that in addition to cells and tissues, exosomes can be contained in fluids such as blood [36], serum [37], amniotic fluid [38], saliva [37], and breast milk [39]. Depending on the cell or tissue type, the secretion of exosomes is regulated by two mechanisms: constitutive mechanism that employs Rab GTPases protein family [40–43] and/or inducible mechanism regulated by several activating factors such as heat shock, hypoxia, DNA damage, increasing of intracellular calcium, and thrombin [40, 44, 45]. Since exosomes have an important role in intercellular communication, they can target a wide variety of cell types at both close and distant target sites to transfer cargo molecules, such as proteins, lipids, and nucleic acids [46]. The proteins that exosomes carry can be MHC I, II [30], tetraspanins such as CD9, CD63, CD81, CD82, CD54, and CD11b [24, 47], HSPs (60, 70, 90) [24, 48], Rab and annexins [49], Clathrin, Alix, and so on [24]. The main biomolecules in the exosome besides proteins are RNAs. To be more specific, in addition to three major RNAs (mRNA, tRNA, and rRNA) involved in protein synthesis, other non-coding RNAs such as miRNAs, small interfering RNAs (siRNAs), circRNA [50], lncRNA [51], small nucleolar RNAs (snoRNAs), and snRNA (all have a vital role in gene expression regulation at both transcriptional and post-transcriptional levels) [52–55] are the main RNAs molecules in the exosomes. Furthermore, piRNAs are also another small non-coding RNA molecules found in exosomes and are resistant to chemical reactions and temperature changes. In animal cells, piRNAs are expressed and assemble into the protein-DNA complex through the Argonaute proteins [56–59]. Many researchers have also found single-stranded [60] and double-stranded DNA within exosomes [61]. Batagov et al. provided evidences that exosomes can transport the 3-UTR fragments of a mRNA, suggesting that these fragments can have a regulatory effect or can be translated into proteins [62].

The following section provides an overview of noncoding RNA nomenclature to help readers understand the terms used in our article. Generally, the homology and function, location, and other factors related to non-coding RNAs contribute to their classification [63]. For example, the name microRNA is indicative of the small size of active RNA molecules, or the name snoRNA, which is an abbreviation for small nucleolar RNA, refers to RNA molecules inside a cell’s nucleolus [64]. Regarding miRNAs, nomenclature can be as follows: a symbol for the human miRNA gene that has been approved by the Human Genome Organization (HUGO) Gene Nomenclature Committee (HGNC) is MIR# format. For example, MIR17 represents a miRNA gene; mir-17 represents a miRNA stem-loop, and miR-17 represents a mature miRNA. Furthermore, each species’ name commences with three letters specific to it. When the letter “has” is used, it refers to humans (Homo sapiens), “hsa-mir-220” as an example, or it refers to rats (Rattus norvegicus) when “rno” is used such as “rno-mir-1”. To identify the members of the same family, the letter is added to the suffix (e.g. hsa-mir-465a and hsa-mir-465b). The 3p’ or 5p’ suffix can also be added if the data are insufficient to determine which sequence is dominant. It is also worth noting that let-7 and lin-4 are notable historical exceptions [64–67].

On the other hand, lncRNA genes consist of a dispersed set of loci related only by their size, which exceed 200 bases, do not have conserved sequence homology, and perform a variety of functions. Generally, it is a known function of the product that determines the naming of lncRNAs. It would be preferable if genes were named based on their normal function rather than mutant phenotypes. Names for genes must be concise and do not try to include all known information about them. As an example, in lncRNA XIST, 'XIST' stands for 'X (inactive)-specific transcript', which is responsible for transcriptionally silencing one of the X chromosomes pairs. No known functions for lncRNA genes are determined by their genomic context [63, 64].

Circulating RNA is generated by the back-splicing of exons from a pre-mRNA, which results in a 3′,5′-phosphodiester bond and creates a circular RNA linked to itself. A nomenclature scheme for cirRNAs has been proposed: “cir[gene symbol]-n”, where the “gene” symbolizes the unspliced host gene, and “n” corresponds to an iterative five-digit number; for example, circPARN-00001 is the first circRNA that has been named for the host gene Poly(A)-specific ribonuclease (*PARN*) [64].

## Exosomes as diagnostic biomarkers

Due to the precise diagnostic and prognostic results provided by exosomes, they have become an interesting research topic in the field of medicine [68–70]. Because exosomes carry biomolecular markers in many types of diseases and are also used as agents to deliver a variety of therapeutic molecules, they can be employed as an efficient tool to detect and treat a wide variety of diseases [71–74]. Besides, there are other advantages of exosomes, including their ease of use, cost-effectiveness, and pain reduction, which allow clinics to consider using exosomes to diagnose many types of diseases, especially cancer, as an alternative to classical surgical methods [75]. Studies that analyze RNAs in exosomes for identifying various types of cancer have been frequently carried out in recent years. For example, Skog et al. examined the serum of patients with glioblastoma and isolated exosomes containing mRNAs and miRNAs. They found that tumor-derived microvesicles are used as a means of transmitting genetic information to the target cells [76]. Takeshita et al. reported that the miR-1246 expression could be used as a prognostic and a diagnostic tool for esophageal squamous cell carcinoma as exosomes could protect miRNA from degradation by RNase [77].

## Overview of NGS

**Fig. 2:**
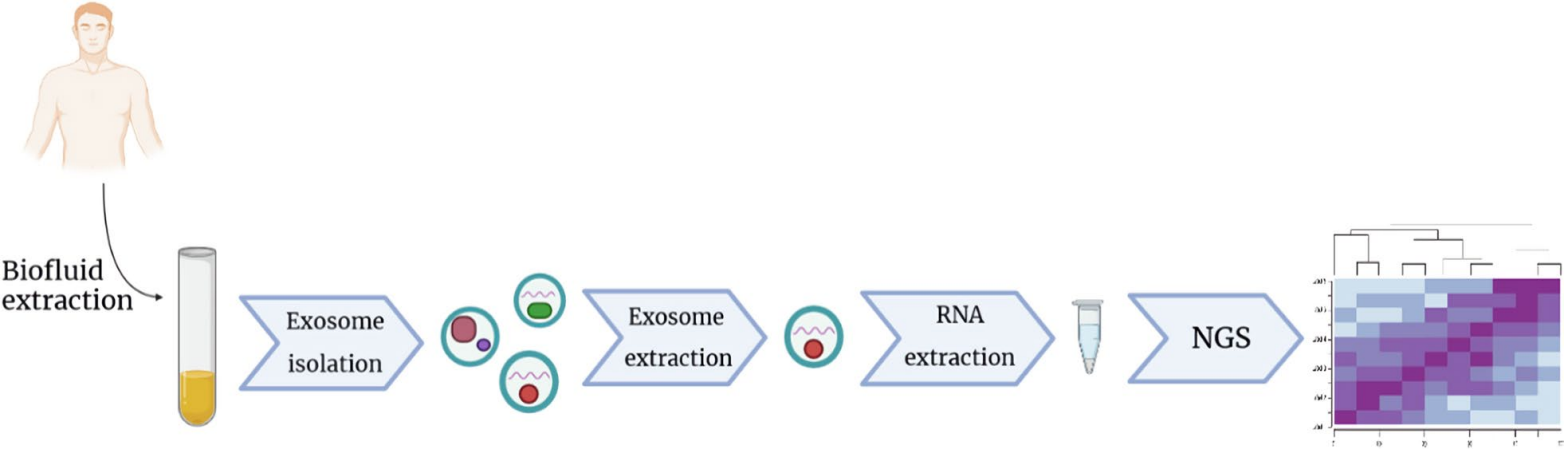
Process of exosomal biomarker identification using NGS. NA: nucleic acid; NGS; next generation sequencing

In the 1990s, many methods were developed for sequencing DNA molecules, and after 2000 the commercialization of NGS platforms was initiated quickly by the companies [78]. This method is a high-throughput sequencing that can perform millions of sequencing reactions simultaneously. NGS is one of the most widely used and advanced tools to identify diseases such as cancer [79, 80]. Using the NGS method, exome, and genome sequencing, chromatin immunoprecipitation sequencing (ChIP-Seq), transcript profiling, and epigenome characterization can be performed [81, 82]. Based on NGS, DNA from cancer samples can be sequenced from a few thousand nucleotides (targeted panel sequencing) to 40–50 million nucleotides (whole-exome sequencing (WES)) and up to 3.3 billion bases (whole-genome sequencing (WGS)).[83]. Currently, targeted gene panels are used in clinics to search for genomic variation in a wide variety of disease-related genes, and whole-exome sequencing is used to identify variation throughout the exome [84]. Over the past few years, NGS has been shown to provide useful information about various cancers and mutations associated with the various types of cancer [85, 86]. Data from the TCGA (The Cancer Genome Atlas), which utilized large-scale genome sequencing to identify markers involved in the 33 cancer types, have contributed to an improvement in cancer diagnosis, treatment, and prevention [87, 88]. Additionally, RNA-sequencing (RNA-seq) has revealed that all forms of RNA, from coding RNA to non-coding RNA, can be detected in exosomes which can be useful for cancer diagnosis [26, 98–103]. Figure 2 illustrates the steps involved in identifying biomarkers of cancer using NGS.

## Potential biomarker: cancer-derived exosomes based on the NGS approach

The following section discusses exosome biomarkers related to various cancer types. Since NGS has become a routine technology in cancer research in recent years and it can provide valuable information about various cancers, the present study attempted to narrow its focus on studies that utilize NGS to analyze exosome noncoding RNAs in cancers. Furthermore, the selections were based on cancers having similar supporting studies.

### Pancreatic cancer

Exosomal components such as miRNAs are used as a screening tool to detect pancreatic malignancies [89, 90]. In this regard Xu et al. collected exosomes from plasma cultures of patients with pancreatic cancer and compared them with healthy individuals, they concluded that pancreatic tumor-derived exosomes were rich in miR-196a (an indicator of pancreatic adenocarcinoma of the pancreas) and miR-1246 (an indicator of intraductal papillary mucinous neoplasms) [91]. Goto et al. studied the exosomal miRNAs of pancreatic cancer patients and found that the expression levels of miR-191, miR-21 (prognostic factors for overall survival), and miR-451(associated with moral nodules) had been increased. Therefore, they suggested that these miRNAs can be used as markers for the growth and metastasis of cancer cells and can be considered as key factors in the early diagnosis of pancreatic cancer [92]. Kumar et al. studied serum exosomes in three groups: healthy individuals, intraductal papillary mucosal neoplasms, and pancreatic ductal adenocarcinoma. They found that there are a wide variety of RNAs such as mRNAs, miRNAs, long intergenic non-coding RNAs (lincRNAs), tRNAs, and piRNAs in the serum exosomes of pancreatic cancer patients [93]. In another study, Chen et al. studied the circRNAs using irradiation on human pancreatic cancer cells and could isolate about 12,572 circRNAs from exosomes of these cells. Among them, they found differentially expressed circRNAs (DE-circRNAs) that were involved in the methylation process [94]. San Lucas et al. examined exosomes from fluid biopsies of patients with pancreaticobiliary cancers and found that exosomal DNAs and RNAs of these patients can be used as acidic nucleic biomarkers to diagnose pancreatic cancer [95].

### Hepatocellular carcinoma

One of the best preconditions to treat hepatocellular carcinoma (HCC) is early and rapid diagnosis because this type of cancer has no special manifestations in the early stages and patients miss the optimal period and timely treatment. Routine screening tests to diagnose the high-risk population of HCC included alpha phytoprotein (AFP) and ultrasonography (US) [96]. Nevertheless, when it comes to the early diagnosis of this type of cancer, exosomes are becoming increasingly important [97, 98]. Many studies have shown that exosomes can serve as a helpful tool for determining the type of tumor, the proliferation rate, and the progression of metastases to specific organs in patients [99]. For example, the Mjelle et al. study which sequenced small RNAs of tissue and serum of HCC patients to evaluate changes in miRNAs expression between different samples showed that miR-21 mediates a direct link between serum exosomes and tumor tissue, allowing miR-21 to enter the bloodstream via exosomes. They also found that other miRNAs such as let-7 were upregulated in serum exosomes in comparison to whole serum, as well as miR-122 quadrupled in the serum of patients with HCC compared with controls [100]. Wang et al. studied exosomal miRNAs in serum to detect HCC, they identified 1244 miRNAs that the expression level of some miRNAs such as miR-122-5p, miR-455-5p, miR-192-5p, miR-100-5p, and miR-1246-5p was almost doubled while, the expression levels of some other miRNAs such as miR-215-5p, miR-4443, miR-486-5p, miR-423-5p, let-7d-3p and miR-203a-3p were reduced more than two times [101]. In another study, Wang et al. found that people with metastatic HCC had high levels of circular RNA-prostaglandin reductase 1 (circPTGR1) expression in their serum exosomes. Through the knockdown of this circRNA, cancer cells can also be stopped from proliferating, migrating, and invading [102]. Jung Cho et al. sequenced miRNAs in exosomes derived from hepatocellular carcinoma and concluded that miR-10b-5p, miR-18a-5p, and miR-940 expression increased in tumor tissues which among them, miR-10b-5p can be used as the main biomarker in the detection of early-stage HCC [103]. Woo et al. examined the onset of pre-metastasis in patients with HCC by sequencing miRNAs in tumor-derived exosomes and found that some miRNAs like miR-1307-5p could enhance the epithelial-to-mesenchymal transition (EMT) process through downregulation of SEC14 like lipid-binding 2 (*SEC14L2*) and Endoglin *(ENG)* genes, therefore miR-1307-5p can be used as a predictive biomarker for HCC metastasis [104]. Yao et al. extracted exosomal lncRNA from the sera of patients with hepatitis, cirrhosis, and HCC, as well as from the sera of healthy individuals, and concluded that lncRNA-family with sequence similarity 72 member D3 (lncRNA-FAM72D-3) and lncRNA- enhancer of polycomb homolog 1–4 (lncRNA-EPC1-4) significantly contribute to hepatocarcinogenesis which can be used as major biomarkers in the diagnosis of HCC [105]. In another study, Huang et al. found several thousand mRNAs and lncRNAs with different expressions in the plasma exosomes of HCC patients. They isolated six differentially expressed lncRNAs from HCC cells, that were involved in processes such as proliferation, migration, and apoptosis. Among them, lncRNA-RP11-85G21.1 (lnc85) was a novel DE‐lncRNA that regulates the function of miR-324-5p and can be considered as a diagnostic biomarker in HCC [106].

### Osteosarcoma (OS)

Exosomes are not only promising targets to detect soft tissue cancers but also they can be easily employed for pursuing cancers such as bone sarcoma [107]. By extracting exosomes from the serum of patients with metastatic osteosarcoma (OS) and sequencing their RNA, Gong et al. found that the expression levels of miR-675 in metastatic OS was increased which subsequently contribute to cell migration and invasion by targeting a migration-related gene Calneuron 1 (*CALN1*) [108]. Jerez et al. identified miRNAs derived from six metastatic osteosarcoma cell lines. Among them, metastatic human osteosarcoma cell line (SAOS2) had the highest exosomal miRNA biomarkers such as miR-21-5p, miR-143-3p, miR-148a-3p, and miR-181a-5p. They also found that these miRNAs may play a key role in the regulation of genes involved in apoptosis and cell adhesion [109]. A recent study by Raimondi et al. analyzed tumor microenvironments associated with OS-derived exosomes and concluded that miR-148a and miR-21-5p are potential tumor diagnostic biomarkers. These miRNAs play a key role in tumor cell growth, osteoclastogenesis, bone resorption, and bone tumor angiogenesis [110]. Based on the molecular profiles of patients with osteosarcomas, Cuscino et al. identified miRNAs in circulating exosomes as biomarkers. There were eight new miRNAs found in three different cell lines that could be used as OS biomarkers, five of which were found to be more prevalent in circulating exosomes among OS patients [111].

### Gastric cancer

Gastric cancer (GC) is the fifth most common and the third deadliest cancer worldwide with more than one million new cases diagnosed each year [112, 113]. Early-stage cancer has a better prognosis, so finding non-invasive biomarkers for GC early detection can be crucial in increasing the survival rate [114]. To date, efforts have been made to evaluate the biomarker potential of exosomal RNAs in gastric cancer. For example, miR-92b-3p, let-7 g-5p, miR-146b-5p, and miR-9-5p are several upregulated miRNAs in GC patients’ exosomes with the biomarker potential. Based on the results, it was observed that these four miRNAs in combination with carcinoembryonic antigen (CEA) have the highest diagnostic value [115]. miR-100 and miR-148a are other upregulated exosomal miRNAs in GC which need to be further investigated as probable biomarkers [116]. Another study has also shown a significant difference in exosomal miRNA expression between gastric cancer stem-like cells (CSCs) and differentiated gastric cancer cells. Thus, exosomal miRNAs evaluation can be informative about GC formation, stage of cancer, and the possibility of recurrence [117]. In a study using unique molecular identifiers (UMI) small RNA sequencing, several dysregulated snRNAs were identified in the serum exosomes from GC patients. Furthermore, the expression levels of miR-1307-3p and piRNAs such as piR-019308, piR-004918, and piR-018569 in GC serum exosomes were significantly higher compared to healthy controls. Therefore, in addition to miRNAs, piRNAs can also be evaluated as biomarkers for GC and GC metastasis [118]. Moreover, in a comparative study between five GC patients and five healthy controls, 620 upregulated and 440 downregulated circRNAs were identified. These circRNAs were involved in important cancer-related signaling pathways. They also had binding sites to interact with miRNAs and take part in expression regulation [119]. With further studies, these RNAs could be used as powerful diagnostic tools or even therapeutic targets for GC.

### Ovarian cancer

Ovarian cancer is one of the most common and lethal cancers among women. Most cases of this cancer are diagnosed in advanced stages which increases the risk of metastasis and greatly reduces the chance of survival. Therefore, finding the right biomarkers to diagnose ovarian cancer as early as possible can have a tremendous impact on the treatment process [120, 121]. So far, relatively few studies have been performed on exosomal RNAs using NGS in ovarian cancer patients. However, more research is being conducted to find possible miRNA biomarkers. A study has shown 34 upregulated and 31 downregulated both in plasma and exosomes, which these miRNAs were previously seen to be involved in cancer-related pathways like the mitogen-activated protein kinase (MAPK) signaling pathway or EMT process [121]. In addition to plasma, another study has been performed on peritoneal exosomes isolated from patients with peritoneal metastatic epithelial ovarian cancer compared with patients with acute pelvic peritonitis as the control group. The results showed that miR-149-3p and miR-222-5p were significantly upregulated and associated with poor survival in epithelial ovarian cancer (EOC). Both of these miRNAs appear to play an oncogenic role in EOC, perhaps through inhibition of MHC I expression [122]. There are also studies of exosomal RNAs that have shown some miRNAs can act as a double-edged sword. For instance, in Yeung et al. study, exosomes derived from cancer-associated adipocytes (CAAs) and cancer-associated fibroblasts (CAFs) revealed the significant abundance of miR-21 in these exosomes [123]. Exosomal miR-21 can be carried to the neighboring cells and spread the malignant phenotype by inducing metastasis. It can also induce chemoresistance by targeting apoptotic protease activating factor 1 (APAF1) [123]. However, on the opposite side, miR-146a and miR-10a which derived from amniotic fluid stem cells (AFSCs) can prevent chemotherapy-induced premature ovarian failure (CTx-induced POF). It has been shown that these two miRNAs exhibit high expression levels in AFSC-derived exosomes in the mouse models. Among these two, it is observed that miR-10a can cause an anti-apoptotic effect in the granulosa cells that have been affected by chemotherapy. Therefore, miR-10a is a possible therapeutic approach for CTx-induced POF [124]. Given what has been discussed, further studies on miRNAs as well as studies on other types of exosomal RNAs could be helpful in the rapid diagnosis and even treatment of ovarian cancer.

### Breast cancer

Breast cancer is the most common cancer among women with an annual increase in incidence. It is the second deadliest cancer after lung cancer. This cancer is highly heterogeneous and complicated. Early detection is crucial to reducing mortality and improving treatment effectiveness [125, 126]. There have been many studies that focused on exosomal microRNAs. For example, a study in 2016 on breast cancer cell lines in mouse models using RNA sequencing showed that the expression levels of miR-1246 and miR-21 were significantly higher in breast cancer exosomes [127]. Additional qRT-PCR studies performed on human patients have shown similar findings [127]. The exosomal miRNAs of plasma samples were analyzed in another study carried out by Wu et al. on 27 patients and 3 healthy controls. Only on triple-negative breast cancer (TNBC) subtype, fifty-four miRNAs (20 upregulated miRNAs and 34 downregulated miRNAs with deregulated expression) were observed, while no significant changes were shown in other subtypes. These fifty-four miRNAs were associated with various cancer-related pathways [128]. Also, a two-year follow-up program showed that the expression levels of hsa-miR-150-5p, hsa-miR-576-3p, and hsa-miR-4665-5p were higher in patients with recurrent breast cancer than in patients with no recurrence. Thus, it is possible that the expression levels of these exosomal miRNAs can help to diagnose recurrent breast cancer [128]. Furthermore, exosomal miRNAs may also be potential biomarkers of cancer metastasis and progression. For instance, miR-363-5p is an exosomal miRNA that is upregulated in breast cancer. It also exhibits a lower expression in patients with lymph node (LN) metastasis and seems to inhibit metastasis via regulating its downstream target platelet-derived growth factor subunit B (PDGFB), thus, this miRNA can be considered as a potential biomarker for axillary lymph node metastasis [129]. Research on exosomes secreted by CAFs has been demonstrated remarkable results. For example, exosomal miR-500a-5p expression is higher in CAFs compared to normal fibroblasts. Exosomes are capable of transmitting this miRNA from CAFs to neighboring cells, allowing it to promote cancer progression and metastasis, and result in an aggressive phenotype in cancer cells [130]. It is also revealed that CD63^+^ CAFs which are associated with tamoxifen resistance in breast cancer patients, secret exosomes with abundant miR-22 [119].

In addition to miRNAs, lncRNAs and circRNAs have been observed to be deregulated in exosomes that originated from breast cancer cells. Evaluation of exosomal lncRNA-H19 in breast cancer patients demonstrated that patients who are resistant to doxorubicin show higher expression levels of exosomal lncRNA-H19 in comparison with parental cells, suggesting that the exosomal lncRNA-H19 can be considered as a biomarker for monitoring doxorubicin resistance in breast cancer [131]. According to research by Dong et al., trastuzumab resistance might be induced in sensitive cells by the release of exosomes secreted by trastuzumab-resistant breast cancer cells that express higher levels of lncRNA-SNHG14 [132]. Besides, the correlation between increased expression of exosomal lncRNA-SNHG14 and distant metastasis and lymph node metastasis of breast cancer has been shown [132]. In addition, it has been found that the expression of exosomal lncRNA-SNHG14 in the serum of patients resistant to trastuzumab was higher when compared with patients who responded to the treatment [132].

Yang et al. investigated the potential clinical application of exosomal circRNAs in the prognosis of TNBC. Their results revealed the differential expression of circRNAs in TNBC cells and non-TNBC cells as well as in their exosomes; they also found that circ-proteasome 20S subunit alpha 1 (circ-PSMA1) act as an upregulated circRNAs in the TNBC cells and their exosomes, as well as in the exosomes isolated from TNBC patients’ serum. Moreover, the results showed that circ-PSMA1 overexpression promoted tumorigenesis, metastasis, and migration in TNBC both in vitro and in vivo [133].

### Prostate cancer

Exosomes are one of the most important biomarkers in the early diagnosis and prognosis of prostate cancer in men [134]. Exosomes can be isolated from the blood or urine of prostate cancer patients and are associated with cancer metastasis [135, 136]. They are involved in RNA exchange between cells that can be used as biomarkers of prostate cancer severity. [137]. Furthermore, Yang et al.'s meta-analysis study confirmed that the exosomal miRNAs can provide a high level of diagnostic information for prostate cancer patients [138]. Over the past several years, researchers have been able to explore and evaluate several important urinary exosomes biomarkers associated with prostate cancer. In this regard, Rodriguez et al. evaluated miRNAs within urinary exosomes as non-invasive biomarkers of prostate cancer. They found that the expression of some miRNAs (such as miR-196a-5p, miR-34a-5p, miR-143-3p, miR-501-3p, and miR-92a-1-5p) was reduced in patients with prostate cancer compared to healthy individuals [139]. Huang et al. found that miR-30a/e-5p isolated from specimens of prostate cancer patients was the best reference control in these patients [140]. Koppers-Lalic et al. evaluated isomiRs (miRNA variants) of urinary exosomes in patients with non-invasive prostate cancer. They found that the expression levels of miR-21, miR-204, and miR-375 increased in cancer patients in comparison to healthy individuals [141]. Furthermore, Zhou et al. found that in patients with prostate cancer, the expression levels of miR-217 (involved in cancer cell proliferation and invasion) and miR-23b-3p (involved in growth inhibition) were increased and decreased, respectively [142]. Huang et al. evaluated the exosomal biomarkers such as miR-1290 and miR-375 from patients with castration-resistant prostate cancer, displayed that these miRNAs were associated with poor overall survival in the screening cohort [140].

## Advantageous

Exosomes offer numerous advantages over other diagnostic methods. Firstly, they are secreted by virtually all living cells, exist in various body fluids, and their overall levels are commonly elevated in most diseases while retaining biological information from the parent cells. These features indicate their importance in reflecting the status of parental cells and the convenience of their extractions [9, 143–149]. For instance, obtaining exosomes from body fluids is more convenient than isolating CTCs as these cells are minimal in the blood [150, 151]. With many known classic extraction approaches and a remarkable number of new methods under development, exosomes are more qualified for clinical application than CTCs [150, 152].

Second, exosomes with lipid bilayers could stabilize and protect biomacromolecules against proteinases, RNases, and other enzymatic activity existing in the biofluids, allowing them to be stored for an extended period unlike various biomarker assays requiring fresh biofluids [153–157]. The storage of exosomes at 4 °C for 24 h followed by freezing at − 80 °C will not change markedly the quality of exosomal markers when compared with immediate storage at − 80 °C and fresh urine samples [158]. Indeed, the high biological stability of exosomes could greatly increase their clinical applications while reducing the cost of sample short-term storage and comforting their transportation [159].

Third, exosomes greatly enhance sensitivity and specificity by reducing the complexity of the biological matrix and reducing noises in the assay, thereby enabling the detection of low abundance biomacromolecules. [160–162]. The amplified sensitivity of exosome-based biomarkers versus total serum and urine biomarkers has been shown by several studies [163–165]. For example, in patients with colorectal cancers, exosomal miRNAs extracted from sera showed higher sensitivity (90%) in comparison to carcinoembryonic antigen (CEA) and carbohydrate antigen 19-9 (CA19-9) serum biomarkers with 30.7% and 16% sensitivity, respectively [165]. In addition, it is challenging to distinguish the noises of normal cells from the signal of cancerous cells while transcriptome analysis of RNA is conducted [166]. Additionally, when RNA is directly isolated from plasma, it is even more challenging to analyze due to the overwhelming megakaryocyte RNA background caused by platelet-derived RNA. [167]. The fact that exosomes contain tumor-specific surface proteins makes them uniquely able to eliminate normal cells noises [166].

Fourth, as far as DNA is concerned, it is more valuable than other sources of DNA, for example, exosomes contain more cfDNA and mitochondrial DNA copies than plasma, which makes them more effective for detecting cancer [168, 169]. Moreover, exosomal DNA mutation frequency has higher detection sensitivity and specificity and greater prognostic value compared with cfDNA [170–173].

Fifth, NGS-based studies have shown that exosomes contain a variety of RNA biotypes such as circRNAs [9] lncRNA [155, 174], mRNAs [76, 175–177], miRNAs [178], mRNA fragments [62], piwi-interacting RNA, and fragments of numerous noncoding RNAs including transfer RNA, ribosomal RNA, Y RNAs, and vault RNA [16, 155, 179–183]. Currently, mRNAs are the most typical biomarkers with clinical applications even though, microRNA studies have dominated the investigations on clinical biomarkers as they are plentiful RNA biotypes with well-known regulatory capabilities (Table 1). The longer RNA biotypes particularly mRNAs with well-known mutations have been known as ‘low hanging fruit’ [9]. Long RNAs not only make the assessment of gene expression levels and the recognition of tumor-specific somatic modifications possible but also make it possible to study the processes indicating disease state or progression [62, 155, 179]. The detection of circRNAs specific to diseases provides effective insights that cannot be obtained by employing small RNA, DNA, or protein assays [9].

**Table 1:** Classification of described exosomal RNA in various cancers

Cancer	Exosomal RNA	Expression levels	Action of mode	Origin of the exosomes	Refs.
Breast cancer	miR-1246, miR-21	Upregulated	–	Plasma (mouse model)	[127]
	miR-363-5p	Downregulated	Inhibition of Axillary lymph node metastasis	Plasma	[129]
	miR-500a-5p	Upregulated	Metastasis	CAFs	[130]
	miR-22	Upregulated	Tamoxifen resistance	CD63 + CAFs	[119]
Breast cancer (TNBC)	miR-150-5p miR-576-3p miR-4665-5p	Upregulated	Cancer recurrence	Plasma	[128]
Gastric cancer	miR-92b-3p, let-7 g-5p, miR-146b-5p, and miR-9-5p	Upregulated	–	Serum	[115]
	miR-100 and miR-148a	Upregulated	–	Gastric cancer cells	[116]
	miR-1290, miR-1246, miR-628-5p, miR-675-3p, miR-424-5p, miR-590-3p	Upregulated	–	CSCs	[117]
	let-7b-5p, miR-224-5p, miR-122-5p, miR-615-3p, miR-5787	Downregulated		CSCs	[117]
	miR-1307-3p, piR-019308, piR-004918, and piR-018569	Upregulated	–	Serum	[118]
	piR-004918 and piR-019308	Upregulated	Metastasis	Serum	[118]
Ovarian cancer	miR-106a-5p, let-7d-5p, and miR-93-5p	Upregulated	–	Plasma	[121]
	miR-122-5p, miR-185-5p, and miR-99b-5p	Downregulated	–	Plasma	[121]
	miR-149-3p and miR-222-5p	Upregulated	–	Peritoneum	[122]
	miR21	Upregulated	Metastasis and chemoresistance	CAAs and CAFs	[123]
Prostate cancer	miR-196a-5p, miR-34a-5p, miR-143-3p, miR-501-3p and miR-92a-1-5p	Downregulated	–	Urine	[139]
	miR-30a/e-5p	Endogenous normalizers	–	Plasma	[140]
	miR-21, miR-204, miR-375	Upregulated	Tumor development	Urine	[141]
	miR-217 miR-23b-3p	Upregulated Downregulated	Cell proliferation, growth inhibition	Plasma	[142]
	miR-1290 and miR-375	Upregulated	–	Plasma	[140]
Pancreatic cancer	miR-196a and miR-1246	Upregulated	Indicator of intraductal papillary mucinous neoplasms and pancreatic adenocarcinoma	Plasma	[91]
	miR-191, miR-21, and miR-451a	Upregulated	Growth and metastasis	Serum	[92]
	miR-21, let-7 and miR-122	Upregulated	–	Tissue and serum	[100]
	miR-122-5p, miR-455-5p, miR-192-5p, miR-100- 5p and miR-1246-5p	Upregulated	Tumor progression	Serum	[101]
Hepatocellular carcinoma	circPTGR1	Upregulated	cell proliferation, migration, and invasion of metastatic tumors	Serum	[102]
	miR-10b-5p, miR-18a-5p and miR-940	Upregulated	Early-stage HCC	Serum	[103]
	miR-1307-5p	Upregulated	Promoting EMT	Serum	[104]
	lnc-FAM72D-3 and lnc-EPC1-4	Upregulated	Tumor cell viability, Inhibition of cell proliferation and cell apoptosis	Serum	[105]
	lnc85	Upregulated	Proliferation, migration, and apoptosis	Plasma	[106]
Osteosarcoma	miR-675	Upregulated	Migration and invasion	Serum	[108]
	miR-21-5p, miR-143-3p, miR-148a-3p, and miR-181a-5p	Upregulated	Metastasis regulation	Serum-free conditioned media	[109]
	miR-148a and miR-21-5p	Upregulated	Bone remodeling and tumor angiogenesis	Low serum medium	[110]

TNBC; triple-negative breast cancer, CAFs; cancer-associated fibroblasts, CSCs; cancer stem-like cells, HCC; hepatocellular carcinoma, EMT; epithelial-to-mesenchymal transition, lncRNA-FAM72D-3; lncRNA-family with sequence similarity 72 member D3, lncRNA-EPC1-4; lncRNA- enhancer of polycomb homolog 1–4

Sixth, exosomes can improve the sensitivity of mutation detection. Compared with ctDNA alone, circulating nucleic acids especially exosomal RNAs could increase the whole number of mutant copies accessible for sampling [9, 171]. Combined analysis of ctDNA and exosomal RNA showed that the epidermal growth factor receptor (EGFR) gene activating mutations had almost 10 times more copies. It suggests that exosomal RNA may increase the potential for detection of mutations in blood samples, particularly when very few copies of ctDNA are available at the beginning of diseases [9, 171]. Moreover, the follow-up of *EGFR*, Kirsten rat sarcoma virus (*KRAS*) and, v-Raf murine sarcoma viral oncogene homolog B1 (*BRAF*) genes mutations in ctDNA and exosomes over time revealed that the combined analysis of tumor materials from exosomes with ctDNA could markedly improve the achievement of a liquid biopsy test [171].

## Limitations

Some obstacles are limiting the clinical application of exosomes as tumor markers. The first limitation is the lack of a rapid, efficient, and cost-effective exosome isolation method, which is essential for reproducible analytical results [157]. Ultracentrifugation, ultrafiltration, density-gradient ultracentrifugation, immunoprecipitation, and size-exclusion chromatography are some of the more common methods used for exosome isolation [184, 185]. Unfortunately, there have been reports that these methods are extremely costly, time-consuming, cumbersome, and they cannot remove impurities [186]. The gold standard for the isolation and purity of exosomes is ultracentrifugation; however, it is the most time-consuming method [3, 187–189]. The second challenge is the identification and quantification of exosomes. Flow cytometry, enzyme-linked immunosorbent, nanoparticle tracking analysis (NTA) are commonly used methods for the quantification of exosomes, each with its own advantages and disadvantages [190]. For example, flow cytometry can detect and sort numerous exosomes by species. However, it requires expensive equipment and yields inconsistent results [3]. Enzyme-linked immunoassay (ELISA) is used for more specific capturing of tumor-derived exosomes by applying specific antibodies against tumor markers such as Glypican-1 (GPC-1), Caveolin-1 (CAV1), or heat shock protein 90 (HSP90) [3]. However, the biological noise or contamination caused by other biomolecules in the sample endangers the qualification of ELISA for exosome identification [3]. NTA could identify the type of exosome, but it is time-consuming as well and requires expensive equipment [3]. An ideal method should address these limitations in terms of purity, cost, equipment, and time to be clinically applicable [3]. Third, the great heterogeneity of extracellular vesicles adds an extra level of difficulty in their purification. There have been many studies developing exosome-based biomarkers, but they have employed different methods for purifying the exosomes, resulting in difficulties with normalizing the results and making comparisons difficult [186]. Despite the argument that exosomes should be classified as blood cells, it has been shown that exosomes have obvious heterogeneity due to their contents as well as their surface characterization [186].

## Conclusion

In recent decades, the failure of conventional solid biopsy in addressing cancer detection has highlighted the importance of liquid biopsy as a minimally invasive and alternative approach for prognosis, diagnosis, and monitoring of cancer patients. A liquid biopsy is acquired from various body fluids and consists of abundant biological information from the originated cells, such as CTC, exosomes, ctDNA, or cell-free DNA, as well as circulating RNAs. The advances in NGS make CTCs and ctDNAs more cost-effective, but they suffer from several limitations, including the limited number of CTCs in the blood or the degradation of ctDNA soon after release. On the other hand, exosomes widely exist in all body fluids, carry about 90% of circulating tumor DNAs, and can protect different biomolecules, such as nucleic acids, proteins, and lipids, against degradation. More importantly, exosomes carrying different biotypes of nucleic acids, reflecting the earlier stages of pathological states, provide a promising platform for cancer prognosis, diagnosis, and the follow-up of treatment in precision and personalized medicine.

Unfortunately, the clinical application of exosomes is currently limited by several factors, including the lack of an ideal isolation and characterization method and the inability to classify exosomes secreted by normal cells and tumor cells. Hence, the need for addressing these limitations to use the unique potential of exosomes for capturing the dynamic complexity of cancer is currently highly demanded.

## Data Availability

Not applicable.

## Abbreviations

EVs: Extracellular vehicles
CTCs: Circulating tumor cells
lncRNA: Long non-coding RNA
tRNA: Transfer RNA
rRNA: Ribosomal RNA
MVBs: Multivesicular bodies
ILVs: Intraluminal vesicles
NGS: Next generation sequencing
MPS: Massively parallel sequencing
WES: Whole-exome
WGS: Whole-genome
HCC: Hepatocellular carcinoma
AFP: Alpha phytoprotein
US: Ultrasonography
OS: Osteosarcoma
GC: Gastric cancer
CSCs: Cancer stem-like cells
UMI: Unique molecular identifiers
circRNA: Circular RNAs
EOC: Epithelial ovarian cancer
CAAs: Cancer-associated adipocytes
AFSCs: Amniotic fluid stem cells
TNBC: Triple-negative breast cancer
LN: Lymph node
PDGFB: Platelet-Derived Growth Factor Subunit B
cfDNA: Cell-free DNA
NTA: Nanoparticle tracking analysis

## References

[1] Kandoth C, McLellan MD, Vandin F, Ye K, Niu B, Lu C, Xie M, Zhang Q (2013). Mutational landscape and significance across 12 major cancer types. Nature.

[2] Egan TK (2000). Monitoring patients undergoing cancer therapy. Lab Med.

[3] Makler A, Asghar W (2020). Exosomal biomarkers for cancer diagnosis and patient monitoring. Expert Rev Mol Diagn.

[4] Lokhandwala T, Bittoni MA, Dann RA, D'Souza AO, Johnson M, Nagy RJ, Lanman RB, Merritt RE (2017). Costs of diagnostic assessment for lung cancer: a medicare claims analysis. Clin Lung Cancer.

[5] Neal R, Tharmanathan P, France B, Din N, Cotton S, Fallon-Ferguson J, Hamilton W, Hendry A (2015). Is increased time to diagnosis and treatment in symptomatic cancer associated with poorer outcomes?. Systematic review Br J Cancer.

[6] Carp LT, Papachristou N, Urch C, Majeed A, El-Khatib M, Aylin P, Atun R, Carp J (2016). Preventing delayed diagnosis of cancer: clinicians’ views on main problems and solutions. J Global Health.

[7] Cassim S, Chepulis L, Keenan R, Kidd J, Firth M, Lawrenson R (2019). Patient and carer perceived barriers to early presentation and diagnosis of lung cancer: a systematic review. BMC Cancer.

[8] Leighl NB, Page RD, Raymond VM, Daniel DB, Divers SG, Reckamp KL, Villalona-Calero MA, Dix D (2019). Clinical utility of comprehensive cell-free DNA analysis to identify genomic biomarkers in patients with newly diagnosed metastatic non–small cell lung cancer. Clin Cancer Res.

[9] Yu W, Hurley J, Roberts D, Chakrabortty S, Enderle D, Noerholm M, Breakefield X, Skog J (2021). Exosome-based liquid biopsies in cancer: opportunities and challenges. Ann Oncol.

[10] Yáñez-Mó M, Siljander PRM, Andreu Z, BedinaZavec A, Borràs FE, Buzas EI, Buzas K, Casal E (2015). Bilogical properties of extracellular vesicles and their physiological functions. J Extracellular Vesicles..

[11] Mathieu M, Martin-Jaular L, Lavieu G, Théry C (2019). Specificities of secretion and uptake of exosomes and other extracellular vesicles for cell-to-cell communication. Nat Cell Biol.

[12] Rak J (2013). Extracellular vesicles–biomarkers and effectors of the cellular interactome in cancer. Front Pharmacol.

[13] Hornick NI, Huan J, Doron B, Goloviznina NA, Lapidus J, Chang BH, Kurre P (2015). Serum exosome microRNA as a minimally-invasive early biomarker of AML. Sci Rep.

[14] Lasda E, Parker R (2016). Circular RNAs co-precipitate with extracellular vesicles: a possible mechanism for circRNA clearance. PLoS ONE.

[15] Ma P, Pan Y, Li W, Sun C, Liu J, Xu T, Shu Y (2017). Extracellular vesicles-mediated noncoding RNAs transfer in cancer. J Hematol Oncol.

[16] NoltetHoen EN, Buermans HP, Waasdorp M, Stoorvogel W, Wauben MH, Hoen PA (2012). Deep sequencing of RNA from immune cell-derived vesicles uncovers the selective incorporation of small non-coding RNA biotypes with potential regulatory functions. Nucleic Acids Res.

[17] Kowal J, Tkach M, Théry C (2014). Biogenesis and secretion of exosomes. Curr Opin Cell Biol.

[18] Brinton LT, Sloane HS, Kester M, Kelly KA (2015). Formation and role of exosomes in cancer. Cell Mol Life Sci.

[19] Harding C, Stahl P (1983). Transferrin recycling in reticulocytes: pH and iron are important determinants of ligand binding and processing. Biochem Biophys Res Commun.

[20] Pan B-T, Johnstone RM (1983). Fate of the transferrin receptor during maturation of sheep reticulocytes in vitro: selective externalization of the receptor. Cell.

[21] Alenquer M, Amorim MJ (2015). Exosome biogenesis, regulation, and function in viral infection. Viruses.

[22] Harding CV, Heuser JE, Stahl PD (2013). Exosomes: looking back three decades and into the future. J Cell Biol.

[23] Théry C, Ostrowski M, Segura E (2009). Membrane vesicles as conveyors of immune responses. Nat Rev Immunol.

[24] Théry C, Zitvogel L, Amigorena S (2002). Exosomes: composition, biogenesis and function. Nat Rev Immunol.

[25] Ge R, Tan E, Sharghi-Namini S, Asada HH (2012). Exosomes in cancer microenvironment and beyond: have we overlooked these extracellular messengers?. Cancer Microenviron..

[26] Pocognoni CA, Berberián MV, Mayorga LS (2015). ESCRT (Endosomal Sorting Complex Required for Transport) machinery is essential for acrosomal exocytosis in human sperm. Biol Reprod.

[27] Christ L, Raiborg C, Wenzel EM, Campsteijn C, Stenmark H (2017). Cellular functions and molecular mechanisms of the ESCRT membrane-scission machinery. Trends Biochem Sci.

[28] Meister M, Bänfer S, Gärtner U, Koskimies J, Amaddii M, Jacob R, Tikkanen R (2017). Regulation of cargo transfer between ESCRT-0 and ESCRT-I complexes by flotillin-1 during endosomal sorting of ubiquitinated cargo. Oncogenesis.

[29] Wollert T, Wunder C, Lippincott-Schwartz J, Hurley JH (2009). Membrane scission by the ESCRT-III complex. Nature.

[30] Beach A, Zhang H-G, Ratajczak MZ, Kakar SS (2014). Exosomes: an overview of biogenesis, composition and role in ovarian cancer. J Ovar Res..

[31] Taylor DD, Gercel-Taylor C (2008). MicroRNA signatures of tumor-derived exosomes as diagnostic biomarkers of ovarian cancer. Gynecol Oncol.

[32] Hu G, Drescher KM, Chen X (2012). Exosomal miRNAs: biological properties and therapeutic potential. Front Genetics..

[33] Théry C, Regnault A, Garin J, Wolfers J, Zitvogel L, Ricciardi-Castagnoli P, Raposo G, Amigorena S (1999). Molecular characterization of dendritic cell-derived exosomes: selective accumulation of the heat shock protein hsc73. J Cell Biol.

[34] Wang G, Dinkins M, He Q, Zhu G, Poirier C, Campbell A, Mayer-Proschel M, Bieberich E (2012). Astrocytes secrete exosomes enriched with proapoptotic ceramide and prostate apoptosis response 4 (PAR-4): potential mechanism of apoptosis induction in Alzheimer disease (AD). J Biol Chem.

[35] Zech D, Rana S, Büchler MW, Zöller M (2012). Tumor-exosomes and leukocyte activation: an ambivalent crosstalk. Cell Commun Signaling..

[36] Baranyai T, Herczeg K, Onódi Z, Voszka I, Módos K, Marton N, Nagy G, Mäger I (2015). Isolation of exosomes from blood plasma: qualitative and quantitative comparison of ultracentrifugation and size exclusion chromatography methods. PLoS ONE.

[37] Gallo A, Tandon M, Alevizos I, Illei GG (2012). The majority of microRNAs detectable in serum and saliva is concentrated in exosomes. PLoS ONE.

[38] Keller S, Ridinger J, Rupp A-K, Janssen JW, Altevogt P (2011). Body fluid derived exosomes as a novel template for clinical diagnostics. J Transl Med.

[39] Qin W, Tsukasaki Y, Dasgupta S, Mukhopadhyay N, Ikebe M, Sauter ER (2016). Exosomes in human breast milk promote EMT. Clin Cancer Res.

[40] Sluijter JP, Verhage V, Deddens JC, van den Akker F, Doevendans PA (2014). Microvesicles and exosomes for intracardiac communication. Cardiovasc Res.

[41] Ostrowski M, Carmo NB, Krumeich S, Fanget I, Raposo G, Savina A, Moita CF, Schauer K (2010). Rab27a and Rab27b control different steps of the exosome secretion pathway. Nat Cell Biol.

[42] Hsu C, Morohashi Y, Yoshimura S-I, Manrique-Hoyos N, Jung S, Lauterbach MA, Bakhti M, Grønborg M (2010). Regulation of exosome secretion by Rab35 and its GTPase-activating proteins TBC1D10A–C. J Cell Biol.

[43] Crenshaw BJ, Sims B, Matthews QL (2018). Biological function of exosomes as diagnostic markers and therapeutic delivery vehicles in carcinogenesis and infectious diseases, in Nanomedicines.

[44] Yu X, Harris SL, Levine AJ (2006). The regulation of exosome secretion: a novel function of the p53 protein. Cancer Res.

[45] Lespagnol A, Duflaut D, Beekman C, Blanc L, Fiucci G, Marine J-C, Vidal M, Amson R (2008). Exosome secretion, including the DNA damage-induced p53-dependent secretory pathway, is severely compromised in TSAP6/Steap3-null mice. Cell Death Differ.

[46] D’Agnelli S, Gerra MC, Bignami E, Arendt-Nielsen L (2020). Exosomes as a new pain biomarker opportunity. Mol Pain.

[47] Villarroya-Beltri C, Baixauli F, Gutiérrez-Vázquez C, Sánchez-Madrid F, Mittelbrunn M. Sorting it out: regulation of exosome loading. In: Seminars in cancer biology. 2014. Elsevier: New York. 10.1016/j.semcancer.2014.04.009 PMC4640178 24769058

[48] Raposo G, Stoorvogel W (2013). Extracellular vesicles: exosomes, microvesicles, and friends. J Cell Biol.

[49] Hessvik NP, Llorente A (2018). Current knowledge on exosome biogenesis and release. Cell Mol Life Sci.

[50] Kristensen LS, Andersen MS, Stagsted LV, Ebbesen KK, Hansen TB, Kjems J (2019). The biogenesis, biology and characterization of circular RNAs. Nat Rev Genet.

[51] Zhao W, Shan B, He D, Cheng Y, Li B, Zhang C, Duan C (2019). Recent progress in characterizing long noncoding RNAs in cancer drug resistance. J Cancer.

[52] Morris KV, Mattick JS (2014). The rise of regulatory RNA. Nat Rev Genet.

[53] Vihinen M (2021). Systematics for types and effects of RNA variations. RNA Biol.

[54] Brosius J, Raabe CA (2016). What is an RNA? A top layer for RNA classification. RNA Biol.

[55] Peng Y, Li J, Zhu L. Chapter 8: Cancer and non-coding RNAs. In:[ BS Ferguson, Nutritional Epigenomics. 2019. New York, Academic Press. p. 119–132.

[56] Wei C. Molecular Biology Select. Cell. 2009;136.

[57] Seto AG, Kingston RE, Lau NC (2007). The coming of age for Piwi proteins. Mol Cell.

[58] Monga I, Banerjee I (2019). Computational identification of piRNAs using features based on rna sequence, structure, thermodynamic and physicochemical properties. Curr Genomics.

[59] Siomi MC, Sato K, Pezic D, Aravin AA (2011). PIWI-interacting small RNAs: the vanguard of genome defence. Nat Rev Mol Cell Biol.

[60] Balaj L, Lessard R, Dai L, Cho Y-J, Pomeroy SL, Breakefield XO, Skog J (2011). Tumour microvesicles contain retrotransposon elements and amplified oncogene sequences. Nat Commun.

[61] Kahlert C, Melo SA, Protopopov A, Tang J, Seth S, Koch M, Zhang J, Weitz J (2014). Identification of double-stranded genomic DNA spanning all chromosomes with mutated KRAS and p53 DNA in the serum exosomes of patients with pancreatic cancer. J Biol Chem.

[62] Batagov AO, Kurochkin IV (2013). Exosomes secreted by human cells transport largely mRNA fragments that are enriched in the 3′-untranslated regions. Biol Direct.

[63] Wright MW (2014). A short guide to long non-coding RNA gene nomenclature. Hum Genomics.

[64] Seal RL, Chen LL, Griffiths-Jones S, Lowe TM, Mathews MB, O'Reilly D, Pierce AJ, Stadler PF (2020). A guide to naming human non-coding RNA genes. EMBO J.

[65] Ambros V, Bartel B, Bartel DP, Burge CB, Carrington JC, Chen X, Dreyfuss G, Eddy SR (2003). A uniform system for microRNA annotation. RNA.

[66] Griffiths-Jones S, Grocock RJ, Van Dongen S, Bateman A, Enright AJ (2006). miRBase: microRNA sequences, targets and gene nomenclature. Nucleic Acids Rese.

[67] Desvignes T, Batzel P, Berezikov E, Eilbeck K, Eppig JT, McAndrews MS, Singer A, Postlethwait J (2015). miRNA nomenclature: a view incorporating genetic origins, biosynthetic pathways, and sequence variants. Trends Genetics..

[68] Dorayappan KDP, Wallbillich JJ, Cohn DE, Selvendiran K (2016). The biological significance and clinical applications of exosomes in ovarian cancer. Gynecol Oncol.

[69] György B, Hung ME, Breakefield XO, Leonard JN (2015). Therapeutic applications of extracellular vesicles: clinical promise and open questions. Annu Rev Pharmacol Toxicol.

[70] Santangelo L, Battistelli C, Montaldo C, Citarella F, Strippoli R, Cicchini C (2017). Functional roles and therapeutic applications of exosomes in hepatocellular carcinoma. BioMed Res Int.

[71] De Toro J, Herschlik L, Waldner C, Mongini C (2015). Emerging roles of exosomes in normal and pathological conditions: new insights for diagnosis and therapeutic applications. Front Immunol.

[72] Lässer C (2015). Exosomes in diagnostic and therapeutic applications: biomarker, vaccine and RNA interference delivery vehicle. Expert Opin Biol Ther.

[73] Wang J, Sun X, Zhao J, Yang Y, Cai X, Xu J, Cao PJF (2017). Exosomes: a novel strategy for treatment and prevention of diseases. Exo.

[74] Norouzi-Barough L, Shirian S, Gorji A, Sadeghi M (2021). Therapeutic potential of mesenchymal stem cell-derived exosomes as a cell-free therapy approach for the treatment of skin, bone, and cartilage defects. Connect Tissue Res.

[75] Vlassov AV, Magdaleno S, Setterquist R, Conrad R (2012). Exosomes: current knowledge of their composition, biological functions, and diagnostic and therapeutic potentials. Biochem Biophys Acta.

[76] Skog J, Würdinger T, Van Rijn S, Meijer DH, Gainche L, Curry WT, Carter BS, Krichevsky AM (2008). Glioblastoma microvesicles transport RNA and proteins that promote tumour growth and provide diagnostic biomarkers. Nat Cell Biol.

[77] Takeshita N, Hoshino I, Mori M, Akutsu Y, Hanari N, Yoneyama Y, Ikeda N, Isozaki Y (2013). Serum microRNA expression profile: miR-1246 as a novel diagnostic and prognostic biomarker for oesophageal squamous cell carcinoma. Br J Cancer.

[78] Franklin WA, Aisner DL, Davies KD, Crooks K, Post MD, Kleinschmidt-DeMasters BK, Ashwood E, Bunn PA (2020). Pathology, biomarkers, and molecular diagnostics. Abeloff's Clinical Oncology.

[79] Shendure J, Ji H (2008). Next-generation DNA sequencing. Nat Biotechnol.

[80] Metzker ML (2010). Sequencing technologies—the next generation. Nat Rev Genet.

[81] De Magalhães JP, Finch CE, Janssens G (2010). Next-generation sequencing in aging research: emerging applications, problems, pitfalls and possible solutions. Ageing Res Rev.

[82] Panahi Y, Fattahi A, Zarei F, Ghasemzadeh N, Mohammadpoor A, Abroon S, Nojadeh JN, Khojastefard M (2018). Next-generation sequencing approaches for the study of genome and epigenome toxicity induced by sulfur mustard. Arch Toxicol.

[83] Haldar A (2021). A K Singh, Next-Generation Sequence Analysis for Clinical Applications. Translational Bioinformatics Applications in Healthcare.

[84] Tucker T, Marra M, Friedman JM (2009). Massively parallel sequencing: the next big thing in genetic medicine. Am J Hum Genet.

[85] Network CGA (2012). Comprehensive molecular portraits of human breast tumours. Nature.

[86] Network CGAR (2012). Comprehensive genomic characterization of squamous cell lung cancers. Nature.

[87] Hoadley KA, Yau C, Wolf DM, Cherniack AD, Tamborero D, Ng S, Leiserson MD, Niu B (2014). Multiplatform analysis of 12 cancer types reveals molecular classification within and across tissues of origin. Cell.

[88] Weinstein JN, Akbani R, Broom BM, Wang W, Verhaak RGW, McConkey D, Lerner S, Morgan M (2014). Comprehensive molecular characterization of urothelial bladder carcinoma. Nature.

[89] Kosaka N (2016). Decoding the secret of cancer by means of extracellular vesicles. J Clin Med.

[90] Hannafon BN, Ding W-Q (2013). Intercellular communication by exosome-derived microRNAs in cancer. Int J Mol Sci.

[91] Xu Y-F, Hannafon BN, Zhao YD, Postier RG, Ding W-Q (2017). Plasma exosome miR-196a and miR-1246 are potential indicators of localized pancreatic cancer. Oncotarget.

[92] Goto T, Fujiya M, Konishi H, Sasajima J, Fujibayashi S, Hayashi A, Utsumi T, Sato H (2018). An elevated expression of serum exosomal microRNA-191,− 21,− 451a of pancreatic neoplasm is considered to be efficient diagnostic marker. BMC Cancer.

[93] Kumar SR, Kimchi ET, Manjunath Y, Gajagowni S, Stuckel AJ, Kaifi JT (2020). RNA cargos in extracellular vesicles derived from blood serum in pancreas associated conditions. Sci Rep.

[94] Chen Y-Y, Jiang M-J, Tian L (2020). Analysis of exosomal circRNAs upon irradiation in pancreatic cancer cell repopulation. BMC Med Genomics.

[95] SanLucas F, Allenson K, Bernard V, Castillo J, Kim D, Ellis K, Ehli E, Davies G (2016). Minimally invasive genomic and transcriptomic profiling of visceral cancers by next-generation sequencing of circulating exosomes. Ann Oncol.

[96] Daniele B, Bencivenga A, Megna AS, Tinessa V (2004). Alpha-fetoprotein and ultrasonography screening for hepatocellular carcinoma. Gastroenterology.

[97] Choi J, Kim GA, Han S, Lee W, Chun S, Lim YS (2019). Longitudinal assessment of three serum biomarkers to detect very early-stage hepatocellular carcinoma. Hepatology.

[98] Galle PR, Foerster F, Kudo M, Chan SL, Llovet JM, Qin S, Schelman WR, Chintharlapalli S (2019). Biology and significance of alpha-fetoprotein in hepatocellular carcinoma. Liver Int.

[99] Hoshino A, Costa-Silva B, Shen T-L, Rodrigues G, Hashimoto A, Mark MT, Molina H, Kohsaka S (2015). Tumour exosome integrins determine organotropic metastasis. Nature.

[100] Mjelle R, Dima SO, Bacalbasa N, Chawla K, Sorop A, Cucu D, Herlea V, Sætrom P (2019). Comprehensive transcriptomic analyses of tissue, serum, and serum exosomes from hepatocellular carcinoma patients. BMC Cancer.

[101] Wang Y, Zhang C, Zhang P, Guo G, Jiang T, Zhao X, Jiang J, Huang X (2018). Serum exosomal micro RNA s combined with alpha-fetoprotein as diagnostic markers of hepatocellular carcinoma. Cancer Med.

[102] Wang G, Liu W, Zou Y, Wang G, Deng Y, Luo J, Zhang Y, Li H (2019). Three isoforms of exosomal circPTGR1 promote hepatocellular carcinoma metastasis via the miR449a–MET pathway. EBioMedicine.

[103] Cho HJ, Eun JW, Baek GO, Seo CW, Ahn HR, Kim SS, Cho SW, Cheong JY (2020). Serum exosomal microRNA, miR-10b-5p, as a potential diagnostic biomarker for early-stage hepatocellular carcinoma. J Clin Med.

[104] Eun JW, Seo CW, Baek GO, Yoon MG, Ahn HR, Son JA, Sung S, Kim DW (2020). Circulating Exosomal MicroRNA-1307–5p as a Predictor for Metastasis in Patients with Hepatocellular Carcinoma. Cancers (Basel)..

[105] Yao Z, Jia C, Tai Y, Liang H, Zhong Z, Xiong Z, Deng M, Zhang Q (2020). Serum exosomal long noncoding RNAs lnc-FAM72D-3 and lnc-EPC1–4 as diagnostic biomarkers for hepatocellular carcinoma. Aging (Albany NY)..

[106] Huang X, Sun L, Wen S, Deng D, Wan F, He X, Tian L, Liang L (2020). RNA sequencing of plasma exosomes revealed novel functional long noncoding RNAs in hepatocellular carcinoma. Cancer Sci.

[107] Brady JV, Troyer RM, Ramsey SA, Leeper H, Yang L, Maier CS, Goodall CP, Ruby CE (2018). A preliminary proteomic investigation of circulating exosomes and discovery of biomarkers associated with the progression of osteosarcoma in a clinical model of spontaneous disease. Transl Oncol.

[108] Gong L, Bao Q, Hu C, Wang J, Zhou Q, Wei L, Tong L, Zhang W (2018). Exosomal miR-675 from metastatic osteosarcoma promotes cell migration and invasion by targeting CALN1. Biochem Biophys Res Commun.

[109] Jerez S, Araya H, Hevia D, Irarrázaval CE, Thaler R, van Wijnen AJ, Galindo M (2019). Extracellular vesicles from osteosarcoma cell lines contain miRNAs associated with cell adhesion and apoptosis. Gene.

[110] Raimondi L, De Luca A, Gallo A, Costa V, Russelli G, Cuscino N, Manno M, Raccosta S (2020). Osteosarcoma cell-derived exosomes affect tumor microenvironment by specific packaging of microRNAs. Carcinogenesis.

[111] Cuscino N, Raimondi L, De Luca A, Carcione C, Russelli G, Conti L, Baldi J, Conaldi PG (2019). Gathering novel circulating exosomal microRNA in osteosarcoma cell lines and possible implications for the disease. Cancers (Basel)..

[112] Thrift AP, El-Serag HB (2020). Burden of Gastric Cancer. Clin Gastroenterol Hepatol.

[113] Xie M, Yu T, Jing X, Ma L, Fan Y, Yang F, Ma P, Jiang H (2020). Exosomal circSHKBP1 promotes gastric cancer progression via regulating the miR-582–3p/HUR/VEGF axis and suppressing HSP90 degradation. Mol Cancer.

[114] Li Q, Shao Y, Zhang X, Zheng T, Miao M, Qin L, Wang B, Ye G (2015). Plasma long noncoding RNA protected by exosomes as a potential stable biomarker for gastric cancer. Tumour Biol.

[115] Tang S, Cheng J, Yao Y, Lou C, Wang L, Huang X, Zhang Y (2020). Combination of Four Serum Exosomal MiRNAs as Novel Diagnostic Biomarkers for Early-Stage Gastric Cancer. Front Genet.

[116] Ren J, Zhou Q, Li H, Li J, Pang L, Su L, Gu Q, Zhu Z (2017). Characterization of exosomal RNAs derived from human gastric cancer cells by deep sequencing. Tumour Biol.

[117] Sun ZP, Li AQ, Jia WH, Ye S, Van Eps G, Yu JM, Yang WJ (2017). MicroRNA expression profiling in exosomes derived from gastric cancer stem-like cells. Oncotarget.

[118] Ge L, Zhang N, Li D, Wu Y, Wang H, Wang J (2020). Circulating exosomal small RNAs are promising non-invasive diagnostic biomarkers for gastric cancer. J Cell Mol Med.

[119] Rao M, Zhu Y, Qi L, Hu F, Gao P (2020). Circular RNA profiling in plasma exosomes from patients with gastric cancer. Oncol Lett.

[120] Giannopoulou L, Zavridou M, Kasimir-Bauer S, Lianidou ES (2019). Liquid biopsy in ovarian cancer: the potential of circulating miRNAs and exosomes. Transl Res.

[121] Zhang H, Xu S, Liu X (2019). MicroRNA profiling of plasma exosomes from patients with ovarian cancer using high-throughput sequencing. Oncol Lett.

[122] Li Y, Liu C, Liao Y, Wang W, Hu B, Lu X, Cui J (2019). Characterizing the landscape of peritoneal exosomal microRNAs in patients with ovarian cancer by high-throughput sequencing. Oncol Lett.

[123] Au Yeung CL, Co NN, Tsuruga T, Yeung TL, Kwan SY, Leung CS, Li Y, Lu ES (2016). Exosomal transfer of stroma-derived miR21 confers paclitaxel resistance in ovarian cancer cells through targeting APAF1. Nat Commun.

[124] Xiao GY, Cheng CC, Chiang YS, Cheng WT, Liu IH, Wu SC (2016). Exosomal miR-10a derived from amniotic fluid stem cells preserves ovarian follicles after chemotherapy. Sci Rep.

[125] Dieterich M, Stubert J, Reimer T, Erickson N, Berling A (2014). Influence of lifestyle factors on breast cancer risk. Breast Care (Basel).

[126] Barrios CH, Reinert T, Werutsky G (2018). Global Breast Cancer Research: Moving Forward. Am Soc Clin Oncol Educ Book.

[127] Hannafon BN, Trigoso YD, Calloway CL, Zhao YD, Lum DH, Welm AL, Zhao ZJ, Blick KE (2016). Plasma exosome microRNAs are indicative of breast cancer. Breast Cancer Res.

[128] Wu H, Wang Q, Zhong H, Li L, Zhang Q, Huang Q, Yu Z (2020). Differentially expressed microRNAs in exosomes of patients with breast cancer revealed by next-generation sequencing. Oncol Rep.

[129] Gao Y, Li X, Zeng C, Liu C, Hao Q, Li W, Zhang K, Zhang W (2020). CD63(+) Cancer-Associated Fibroblasts Confer Tamoxifen Resistance to Breast Cancer Cells through Exosomal miR-22. Adv Sci (Weinh)..

[130] Chen B, Sang Y, Song X, Zhang D, Wang L, Zhao W, Liang Y, Zhang N (2021). Exosomal miR-500a-5p derived from cancer-associated fibroblasts promotes breast cancer cell proliferation and metastasis through targeting USP28. Theranostics.

[131] Wang X, Pei X, Guo G, Qian X, Dou D, Zhang Z, Xu X, Duan X (2020). Exosome-mediated transfer of long noncoding RNA H19 induces doxorubicin resistance in breast cancer. J Cell Physiol.

[132] Dong H, Wang W, Chen R, Zhang Y, Zou K, Ye M, He X, Zhang F (2018). Exosome-mediated transfer of lncRNA-SNHG14 promotes trastuzumab chemoresistance in breast cancer. Int J Oncol.

[133] Yang S-J, Wang D-D, Zhong S-L, Chen W-Q, Wang F-L, Zhang J, Xu W-X, Xu D (2021). Tumor-derived exosomal circPSMA1 facilitates the tumorigenesis, metastasis, and migration in triple-negative breast cancer (TNBC) through miR-637/Akt1/β-catenin (cyclin D1) axis. Cell Death Dis.

[134] Vlaeminck-Guillem V (2018). Extracellular vesicles in prostate cancer carcinogenesis, diagnosis, and management. Front Oncol.

[135] Tavoosidana G, Ronquist G, Darmanis S, Yan J, Carlsson L, Wu D, Conze T, Ek P (2011). Multiple recognition assay reveals prostasomes as promising plasma biomarkers for prostate cancer. Proc Natl Acad Sci.

[136] Øverbye A, Skotland T, Koehler CJ, Thiede B, Seierstad T, Berge V, Sandvig K, Llorente A (2015). Identification of prostate cancer biomarkers in urinary exosomes. Oncotarget.

[137] Soekmadji C, Russell PJ, Nelson CC (2013). Exosomes in prostate cancer: putting together the pieces of a puzzle. Cancers (Basel)..

[138] Yang B, Xiong W-Y, Hou H-J, Xu Q, Cai X-L, Zeng T-X, Ha X-Q (2019). Exosomal miRNAs as Biomarkers of Cancer: a Meta-Analysis. Clin Lab.

[139] Rodríguez M, Bajo-Santos C, Hessvik NP, Lorenz S, Fromm B, Berge V, Sandvig K, Linē A (2017). Identification of non-invasive miRNAs biomarkers for prostate cancer by deep sequencing analysis of urinary exosomes. Mol Cancer.

[140] Huang X, Yuan T, Liang M, Du M, Xia S, Dittmar R, Wang D, See W (2015). Exosomal miR-1290 and miR-375 as prognostic markers in castration-resistant prostate cancer. Eur Urol.

[141] Koppers-Lalic D, Hackenberg M, De Menezes R, Misovic B, Wachalska M, Geldof A, Zini N, De Reijke T (2016). Non-invasive prostate cancer detection by measuring miRNA variants (isomiRs) in urine extracellular vesicles. Oncotarget.

[142] Zhou C, Chen Y, He X, Zheng Z, Xue D (2020). Functional Implication of Exosomal miR-217 and miR-23b-3p in the Progression of Prostate Cancer. Onco Targets Ther.

[143] Buzas EI, György B, Nagy G, Falus A, Gay S (2014). Emerging role of extracellular vesicles in inflammatory diseases. Nat Rev Rheumatol.

[144] King HW, Michael MZ, Gleadle JM (2012). Hypoxic enhancement of exosome release by breast cancer cells. BMC Cancer.

[145] Momen-Heravi F, Saha B, Kodys K, Catalano D, Satishchandran A, Szabo G (2015). Increased number of circulating exosomes and their microRNA cargos are potential novel biomarkers in alcoholic hepatitis. J Transl Med.

[146] Akers JC, Ramakrishnan V, Kim R, Skog J, Nakano I, Pingle S, Kalinina J, Hua W (2013). MiR-21 in the extracellular vesicles (EVs) of cerebrospinal fluid (CSF): a platform for glioblastoma biomarker development. PLoS ONE.

[147] Boukouris S, Mathivanan S (2015). Exosomes in bodily fluids are a highly stable resource of disease biomarkers. Proteom Clin Appl..

[148] Turchinovich A, Weiz L, Langheinz A, Burwinkel B (2011). Characterization of extracellular circulating microRNA. Nucleic Acids Res.

[149] Cai X, Janku F, Zhan Q, Fan J-B (2015). Accessing genetic information with liquid biopsies. Trends Genet.

[150] Tayoun T, Faugeroux V, Oulhen M, Aberlenc A, Pawlikowska P, Farace F (2019). CTC-derived models: a window into the seeding capacity of circulating tumor cells (CTCs). Cells.

[151] Cubero MA (2017). J Lorente, I Robles-Fernandez, A Rodriguez-Martinez, J Puche, M Serrano, *Circulating tumor cells: markers and methodologies for enrichment and detection*. Circulating Tumor Cells.

[152] Théry C, Amigorena S, Raposo G, Clayton A (2006). Isolation and characterization of exosomes from cell culture supernatants and biological fluids. Curr Protoc Cell Biol.

[153] Kalra H, Adda CG, Liem M, Ang CS, Mechler A, Simpson RJ, Hulett MD, Mathivanan S (2013). Comparative proteomics evaluation of plasma exosome isolation techniques and assessment of the stability of exosomes in normal human blood plasma. Proteomics.

[154] Momen-Heravi F, Bala S, Bukong T, Szabo G (2014). Exosome-mediated delivery of functionally active miRNA-155 inhibitor to macrophages. Nanomed Nanotechnol Biol Med.

[155] Huang X, Yuan T, Tschannen M, Sun Z, Jacob H, Du M, Liang M, Dittmar RL (2013). Characterization of human plasma-derived exosomal RNAs by deep sequencing. BMC Genomics.

[156] Momen-Heravi F, Bala S, Kodys K, Szabo G (2015). Exosomes derived from alcohol-treated hepatocytes horizontally transfer liver specific miRNA-122 and sensitize monocytes to LPS. Sci Rep.

[157] Momen-Heravi F, Getting SJ, Moschos SA (2018). Extracellular vesicles and their nucleic acids for biomarker discovery. Pharmacol Ther.

[158] Cheruvanky A, Zhou H, Pisitkun T, Kopp JB, Knepper MA, Yuen PS, Star RA (2007). Rapid isolation of urinary exosomal biomarkers using a nanomembrane ultrafiltration concentrator. Am J Physiol Renal Physiol.

[159] Zhou B, Xu K (2020). Application of exosomes as liquid biopsy in clinical diagnosis. Clinical.

[160] Boukouris S, Mathivanan S (2015). Exosomes in bodily fluids are a highly stable resource of disease biomarkers. Proteomics Clin Appl.

[161] Willis JC, Lord GM (2015). Immune biomarkers: the promises and pitfalls of personalized medicine. Nat Rev Immunol.

[162] Momen-Heravi F, Saha B, Kodys K, Catalano D, Satishchandran A, Szabo G (2015). Increased number of circulating exosomes and their microRNA cargos are potential novel biomarkers in alcoholic hepatitis. J Transl Med.

[163] Logozzi M, De Milito A, Lugini L, Borghi M, Calabrò L, Spada M, Perdicchio M, Marino ML (2009). High levels of exosomes expressing CD63 and caveolin-1 in plasma of melanoma patients. PLoS ONE.

[164] Madhavan B, Yue S, Galli U, Rana S, Gross W, Müller M, Giese NA, Kalthoff H (2015). Combined evaluation of a panel of protein and miRNA serum-exosome biomarkers for pancreatic cancer diagnosis increases sensitivity and specificity. Int J Cancer.

[165] Ogata-Kawata H, Izumiya M, Kurioka D, Honma Y, Yamada Y, Furuta K, Gunji T, Ohta H (2014). Circulating exosomal microRNAs as biomarkers of colon cancer. PLoS ONE.

[166] Yu W, Hurley J, Roberts D, Chakrabortty SK, Enderle D, Noerholm M, Breakefield XO, Skog JK (2021). Exosome-based liquid biopsies in cancer: opportunities and challenges. Ann Oncol.

[167] Brinkman K, Meyer L, Bickel A, Enderle D, Berking C, Skog J, Noerholm M (2020). Extracellular vesicles from plasma have higher tumour RNA fraction than platelets. J Extracell Vesicles..

[168] Fernando MR, Jiang C, Krzyzanowski GD, Ryan WL (2017). New evidence that a large proportion of human blood plasma cell-free DNA is localized in exosomes. PLoS ONE.

[169] Keserű JS, Soltész B, Lukács J, Márton É, Szilágyi-Bónizs M, Penyige A, Póka R, Nagy B (2019). Detection of cell-free, exosomal and whole blood mitochondrial DNA copy number in plasma or whole blood of patients with serous epithelial ovarian cancer. J Biotechnol.

[170] Allenson K, Castillo J, SanLucas FA, Scelo G, Kim DU, Bernard V, Davis G, Kumar T (2017). High prevalence of mutant KRAS in circulating exosome-derived DNA from early-stage pancreatic cancer patients. Ann Oncol.

[171] Möhrmann L, Huang HJ, Hong DS, Tsimberidou AM, Fu S, Piha-Paul SA, Subbiah V, Karp DD (2018). Liquid biopsies using plasma exosomal nucleic acids and plasma cell-free DNA compared with clinical outcomes of patients with advanced cancers. Clin Cancer Res.

[172] Wan Y, Liu B, Lei H, Zhang B, Wang Y, Huang H, Chen S, Feng Y (2018). Nanoscale extracellular vesicle-derived DNA is superior to circulating cell-free DNA for mutation detection in early-stage non-small-cell lung cancer. Ann Oncol.

[173] Bernard V, Kim DU, San Lucas FA, Castillo J, Allenson K, Mulu FC, Stephens BM, Huang J (2019). Circulating nucleic acids are associated with outcomes of patients with pancreatic cancer. Gastroenterology.

[174] Kogure T, Yan IK, Lin W-L, Patel T (2013). Extracellular vesicle–mediated transfer of a novel long noncoding RNA TUC339: a mechanism of intercellular signaling in human hepatocellular cancer. Genes Cancer.

[175] Baj-Krzyworzeka M, Szatanek R, Węglarczyk K, Baran J, Urbanowicz B, Brański P, Ratajczak MZ, Zembala M (2006). Tumour-derived microvesicles carry several surface determinants and mRNA of tumour cells and transfer some of these determinants to monocytes. Cancer Immunol, Immunother.

[176] Bruno S, Grange C, Collino F, Deregibus MC, Cantaluppi V, Biancone L, Tetta C, Camussi G (2012). Microvesicles derived from mesenchymal stem cells enhance survival in a lethal model of acute kidney injury. PLoS ONE.

[177] Deregibus MC, Cantaluppi V, Calogero R, LoIacono M, Tetta C, Biancone L, Bruno S, Bussolati B (2007). Endothelial progenitor cell–derived microvesicles activate an angiogenic program in endothelial cells by a horizontal transfer of mRNA. Blood.

[178] Huang Q, Yang J, Zheng J, Hsueh C, Guo Y, Zhou L (2018). Characterization of selective exosomal microRNA expression profile derived from laryngeal squamous cell carcinoma detected by next generation sequencing. Oncol Rep.

[179] Miranda KC, Bond DT, Levin JZ, Adiconis X, Sivachenko A, Russ C, Brown D, Nusbaum C (2014). Massively parallel sequencing of human urinary exosome/microvesicle RNA reveals a predominance of non-coding RNA. PLoS ONE.

[180] Bellingham SA, Coleman BM, Hill AF (2012). Small RNA deep sequencing reveals a distinct miRNA signature released in exosomes from prion-infected neuronal cells. Nucleic Acids Res.

[181] Dhahbi JM, Spindler SR, Atamna H, Boffelli D, Martin DI (2014). Deep sequencing of serum small RNAs identifies patterns of 5′ tRNA half and YRNA fragment expression associated with breast cancer. Biomark Cancer..

[182] Dhahbi JM, Spindler SR, Atamna H, Boffelli D, Mote P, Martin DI (2013). 5′-YRNA fragments derived by processing of transcripts from specific YRNA genes and pseudogenes are abundant in human serum and plasma. Physiol Genomics.

[183] Freedman JE, Gerstein M, Mick E, Rozowsky J, Levy D, Kitchen R, Das S, Shah R (2016). Diverse human extracellular RNAs are widely detected in human plasma. Nat Commun.

[184] Li P (2017). M Kaslan, S H Lee, J Yao, Z Gao. Progress in exosome isolation techniques Theranostics.

[185] Greening DW (2015). R Xu, H Ji, B J Tauro, R J Simpson, A protocol for exosome isolation and characterization: evaluation of ultracentrifugation, density-gradient separation, and immunoaffinity capture methods. Proteomic Profiling.

[186] Xiao Y, Zhong J, Zhong B, Huang J, Jiang L, Jiang Y, Yuan J, Sun J (2020). Exosomes as potential sources of biomarkers in colorectal cancer. Cancer Lett.

[187] Konoshenko MY, Lekchnov EA, Vlassov AV, Laktionov PP (2018). Isolation of extracellular vesicles: general methodologies and latest trends. BioMed Res Int.

[188] Gupta S, Rawat S, Arora V, Kottarath SK, Dinda AK, Vaishnav PK, Nayak B, Mohanty S (2018). An improvised one-step sucrose cushion ultracentrifugation method for exosome isolation from culture supernatants of mesenchymal stem cells. Stem Cell Res Ther.

[189] Koh YQ, Almughlliq FB, Vaswani K, Peiris HN, Mitchell MD (2018). Exosome enrichment by ultracentrifugation and size exclusion chromatography. Front Biosci (Landmark Ed)..

[190] Espinal AE, Yan Y, Zhang L, Espinal L, Morey A, Wells BO, Aindow M, Suib SL (2014). Substrate control of anisotropic resistivity in heteroepitaxial nanostructured arrays of cryptomelane manganese oxide on strontium titanate. Small.

